# Boundary extension is attenuated in patients with ventromedial prefrontal cortex damage

**DOI:** 10.1016/j.cortex.2018.07.002

**Published:** 2018-11

**Authors:** Flavia De Luca, Cornelia McCormick, Sinead L. Mullally, Helene Intraub, Eleanor A. Maguire, Elisa Ciaramelli

**Affiliations:** a Dipartimento di Psicologia and Centro studi e ricerche di Neuroscienze Cognitive, Università di Bologna, Bologna, Italy; b Wellcome Centre for Human Neuroimaging, Institute of Neurology, University College London, London, UK; c Institute of Neuroscience, Newcastle University, Newcastle, UK; d Department of Psychological and Brain Sciences, University of Delaware, Newark, USA

**Keywords:** Scene construction, Event construction, Hippocampus, vmPFC, Boundary extension

## Abstract

The ventromedial prefrontal cortex (vmPFC) and hippocampus have been implicated in the mental construction of scenes and events. However, little is known about their specific contributions to these cognitive functions. Boundary extension (BE) is a robust indicator of fast, automatic, and implicit scene construction. BE occurs when individuals who are viewing scenes automatically imagine what might be beyond the view, and consequently later misremember having seen a greater expanse of the scene. Patients with hippocampal damage show attenuated BE because of their scene construction impairment. In the current study, we administered BE tasks to patients with vmPFC damage, brain-damaged control patients, and healthy control participants. We also contrasted the performance of these patients to the previously-published data from patients with hippocampal lesions (Mullally, Intraub, & Maguire, 2012). We found that vmPFC-damaged patients showed reduced BE compared to brain-damaged and healthy controls. Indeed, BE attenuation was similar following vmPFC or hippocampal damage. Notably, however, whereas hippocampal damage seems to particularly impair the spatial coherence of scenes, vmPFC damage leads to a difficulty constructing scenes in a broader sense, with the prediction of what should be in a scene, and the monitoring or integration of the scene elements being particularly compromised. We conclude that vmPFC and hippocampus play important and complementary roles in scene construction.

## 1 Introduction

For most of us, if we close our eyes we can construct vivid mental scenes and events that help us to remember the past, envision the future and create fictitious scenarios. Neuroimaging and neuropsychological evidence has pinpointed several key brain regions that seem to support these functions, including the ventromedial prefrontal cortex (vmPFC) and hippocampus (Addis et al., 2007, Bertossi et al., 2016a, Bertossi et al., 2016b, Hassabis et al., 2007, Lah and Miller, 2008, Race et al., 2011, Svoboda et al., 2006). However, the separate contributions of vmPFC and hippocampus are not well understood. One way to try and dissociate the roles of these two brain regions is to administer tasks that have been associated with one brain structure to patients with damage to the other brain structure. Emerging evidence suggests that the hippocampus is necessary for constructing mental models of spatially-coherent scenes in which details can be bound in order to be re- or pre-experienced (Maguire and Mullally, 2013, Clark and Maguire, 2016, Zeidman and Maguire, 2016; McCormick, Ciaramelli, De Luca, & Maguire, 2018).

In this regard, an especially intriguing scene construction phenomenon is “boundary extension” (BE) (Intraub and Richardson, 1989, Intraub, 2012). BE occurs when individuals who are viewing scenes automatically imagine what might be beyond the view, and consequently later misremember having seen a greater expanse of the scene. BE is a powerful psychological phenomenon that has been replicated in many healthy populations, including adults (Chadwick et al., 2013, Intraub and Richardson, 1989, Intraub et al., 1992, Intraub et al., 1998), children (Kreindel and Intraub, 2017, Seamon et al., 2002), babies (Quinn & Intraub, 2007), and also cohorts with developmental disorders (Spanò, Intraub, & Edgin, 2017). What makes BE so intriguing is that healthy participants tend to make robust and confident memory errors despite seeing the original scenes just a few milliseconds beforehand. Therefore, BE provides a unique window into the implicit, automatic and fast process of internal scene construction.

Patients with bilateral hippocampal damage, who have impaired scene construction ability, show attenuated BE, leading to paradoxically better memory performance compared to control participants despite their amnesia (Mullally, Intraub, & Maguire, 2012). This is because when processing the scenes, the patients do so without using scene construction to extrapolate beyond the scene boundaries. When BE was examined using functional magnetic resonance imaging (fMRI), the importance of the hippocampus was further underlined (Chadwick et al., 2013; see also Park, Intraub, Yi, Widders, & Chun, 2007). Interestingly, effective connectivity analyses during BE showed that the hippocampus influenced activity in the visual-perceptual cortices rather than vice versa. This finding highlights one of the pathways underpinning this top-down process.

Whereas the previous fMRI study did not reveal any prefrontal cortex activation, the vmPFC has recently been implicated in top-down initiation of hippocampal processes (Garrido, Barnes, Kumaran, Maguire, & Dolan, 2015; for reviews, see (McCormick, Ciaramelli, et al., 2018a; Moscovitch, Cabeza, Winocur, & Nadel, 2016). In addition, vmPFC-damaged patients are impaired at imagining future and fictitious events compared to control groups, and rate their constructed experiences as lacking spatial coherence (Bertossi et al., 2016a). Together, these findings suggest that the vmPFC plays a role in mental scene construction, at least for tasks that are explicit and involve introspection.

The question we address here is whether vmPFC is equally involved in the fast, implicit and automatic process of BE. We investigated BE in patients with vmPFC lesions and also control patients with cortical lesions that did not involve the vmPFC. In addition, we contrasted their performance with the hippocampal patients described by Mullally et al. (2012). If vmPFC is necessary for scene construction, patients with vmPFC damage should show reduced BE compared to healthy and brain-damaged controls, similar to hippocampal-damaged patients. This would confirm the role of vmPFC in scene construction using a paradigm that probes construction implicitly, and in relation to single scenes. An additional 'scene probe' task allowed us to interrogate scene construction further in terms of perceptual, emotional, and spatial details, providing insight into the nature of the impairment associated with damage to the vmPFC or the hippocampus.

## 2 Methods

### 2.1 Participants

Twenty-five patients took part in the experiment. Eight patients had vmPFC damage (vmPFC patients; all males, mean age = 59.25 years, range = 46–72; see Table 1 for demographic and clinical information), and ten 'control' patients had brain damage that did not involve vmPFC or the hippocampus (control patients; seven males, mean age = 59.30 years, range = 45–67 years; Table 1). We also considered data from seven patients who had hippocampal damage (hippocampal patients; four males, mean age = 41.43 years, range = 32–63 years). vmPFC and control patients were Italian and were recruited at the Centre for Studies and Research in Cognitive Neuroscience, Cesena, Italy. Hippocampal patients were British, and were tested at the Wellcome Centre for Human Neuroimaging, University College London, UK. The background details of the hippocampal patients and their scores on the BE tasks have already been reported in Mullally et al. (2012) (for convenience, background details are summarized in Supplementary Materials Table S1). Our main interest here was in the new data relating to performance of the vmPFC and control patients. We reprise relevant data from the hippocampal patients to afford direct comparisons with the vmPFC and control patients as an interesting secondary analysis.

**Table 1:** Demographic and clinical data of vmPFC patients, control patients, and vmPFC healthy controls.

	N	Gender	Age (years)	Edu (years)	SPM	Phonemic fluency	Semantic fluency	Digit span	Corsi test	Prose recall	ROCF copy	ROCF recall
vmPFC patients	8	8 M	59.25 (8.60)	9.88 (3.04)	28.84 (4.35)	24.75 (9.39)	38.25 (13.60)	5.50 (.79)	4.48 (.82)	8.27 (4.09)	31.28 (5.05)	13.63 (5.54)
Control patients	10	3 F, 7 M	59.30 (8.81)	9.50 (3.14)	24.78 (7.96)	32.00 (7.60)	48.40 (11.36)	5.43 (.92)	4.03 (1.23)	11.75 (2.36)	29.22 (8.25)	13.08 (8.65)
vmPFC healthy controls	10	10 M	56.50 (5.70)	9.00 (2.11)	–	–	–	–	–	–	–	–

Notes. vmPFC = ventromedial prefrontal cortex; M = male; F = female; Edu = education; SPM = Raven's Standard Progressive Matrices; ROCF = Rey–Osterrieth Complex Figure. We report mean corrected scores (see Spinnler & Tognoni, 1987 for normative data), in all cases within the normal limits, and the standard deviation of the mean in parentheses.

Brain damage in vmPFC patients was bilateral in all cases, and the result of the rupture of an aneurysm of the anterior communicating artery (ACoA). In control patients, brain damage (left hemisphere: five cases; right hemisphere: five cases) was due to stroke (six cases), arteriovenous malformations (two cases), or tumor (two cases). Lesion sites in control patients included the occipital cortex and occipital-temporal area, and lateral aspects of the temporal and prefrontal cortex (see Supplementary Figure S1). The hippocampal-damaged patients’ lesions were focal and also bilateral (Supplementary Materials Table S1; Figure S2; full details in Mullally et al., 2012). All patients were in a stable phase of recovery (at least 3 months post-lesion) and had no other diagnoses likely to affect cognition or interfere with participation in the study (e.g., significant psychiatric disease, alcohol abuse, history of cerebrovascular disease).

Ten Italian healthy individuals (vmPFC healthy controls; all males; mean age = 56.50 years, range = 44–63; see Table 1) were matched to the vmPFC and control patients on age (F_(2,25)_ = .41, *p* = .67), education (F_(2,25)_ = .22, *p* = .80), and gender balance (vmPFC: *χ*^2^ = .00, *p* = 1.00; control patients: *χ*^2^ = 1.09, *p* = .30). The hippocampal patients were generally younger than the vmPFC and control patients (F_(2,22)_ = 9.77, *p* = .001), and so were matched with twelve British healthy individuals (eight males, mean age = 42.67 years, range = 32–63; Supplementary Materials Table S1; see Mullally et al., 2012) on age (U = 34, Z = −.68, *p* = .496), gender balance (*χ*^2^ = .17, *p* = .68), and IQ (U = 22.5, Z = −1.66, *p* = .097). Healthy control participants were not taking psychoactive drugs and were free of current or past psychiatric or neurological illness as determined by history. These sample sizes were chosen based on the previous neuropsychological study that examined BE (Mullally et al., 2012). Participants gave informed consent in accordance with the Bioethical Committee of the University of Bologna, the CEIIAV Ethical Committee of Emilia Romagna Regional Health Service, and the National Research Ethics Committee (London, Queen Square, UK), and in line with the Declaration of Helsinki (Editors, 1991).

#### 2.1.1 Neuropsychological profile

Table 1 shows the vmPFC and control patients' neuropsychological profiles. In general the patients’ cognitive functioning was preserved, as indicated by their scores on the Raven Standard Progressive Matrices (see Spinnler & Tognoni, 1987, for normative data) and verbal fluency (Spinnler & Tognoni, 1987), which were within the normal range in both groups. vmPFC and control patients also had intact verbal and spatial short-term memory, as assessed with the digit span and Corsi tests (Spinnler & Tognoni, 1987) and verbal and spatial long-term memory, as assessed with prose recall and recall of the Rey–Osterrieth complex figure (Spinnler & Tognoni, 1987). The copy of the Rey–Osterrieth complex figure was also normal (Spinnler & Tognoni, 1987). Direct comparison of the vmPFC patients and control patients showed comparable scores in the above neuropsychological tests (*p* > .09 in all cases) with the exception of prose recall, which was poorer in vmPFC compared to control patients (t = −2.18, *p* = .045). In some cases, control patients with posterior lesions had visual field deficits, including hemianopia (in six cases) and quadrantopia (in one case). In these patients, however, detection of visual stimuli in standardized tests was not impaired when eye movements were allowed, and visual search performance was within the normal limits (see Supplementary Table S2). We therefore assumed they would be able to perform the BE task (see below). The hippocampal patients were high functioning and did not have any cognitive impairments other than severe memory deficits (see Supplementary Materials Table S1; Mullally et al., 2012).

#### 2.1.2 Lesion analysis

vmPFC and control patients’ individual lesions, derived from magnetic resonance imaging or computerized tomography images, were manually drawn by an expert neurologist (not involved in the present study, and blind to task performance), or by F.D.L, and then verified by the same neurologist, directly on each slice of the normalized T1-weighted template MRI scan from the Montreal Neurological Institute (Holmes et al., 1998). This template is approximately oriented to match Talairach space (Talairach & Tournoux, 1988) and is distributed with MRIcro (Holmes et al., 1998). This manual procedure combines segmentation (identification of lesion boundaries) and registration (to a standard template) into a single step, with no additional transformation required (Kimberg, Coslett, & Schwartz, 2007). MRIcro software was used to estimate lesion volumes (in cc) and generate lesion overlap images.

Fig. 1 shows the extent and overlap of brain lesions in vmPFC patients. The Brodmann areas (BA) that were mainly affected were BA 10, BA 11, BA 24, BA 25, BA 32, with the region of maximal overlap occurring in BA 11 (M = 17.30 cc, SD = 9.85), BA 10 (M = 8.79 cc, SD = 6.65), and BA 32 (M = 6.06 cc, SD = 3.34). One vmPFC patient had a very large lesion that extended to dorsal prefrontal cortex (BA 6 and BA 8). Excluding this patient from the analyses, however, did not alter the results. For the control patients, the areas mainly affected were BAs 17–19 (M = 5.68 cc, SD = 9.08), BAs 20–22 and BA 37 (M = 14.06 cc, SD = 20.81), and BAs 39–40 (M = 2.90 cc, SD = 5.58). There was no significant difference in lesion volume between vmPFC patients and control patients (42.61 *vs*. 40.38 cc, t = .12, *p* = .91). The hippocampal patients had selective bilateral hippocampal damage as confirmed by manual segmentation of the hippocampi and automated whole-brain grey matter analyses as described in Mullally et al. (2012).

**Fig. 1:**
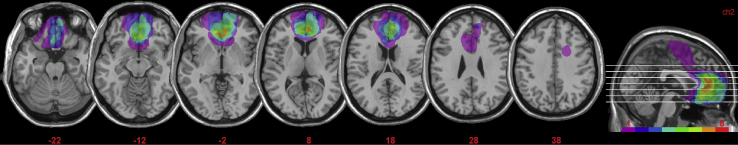
Representative axial slices and cumulative midsagittal view of the standard Montreal Neurological Institute brain showing the extent of lesion overlap in the vmPFC patients. The white horizontal lines on the sagittal view are the positions of the axial slices, and the red numbers below the axial views are the z coordinates of each slice. The color bar indicates the number of overlapping lesions. Maximal overlap occurs in BA 10, 11, and 32. The left hemisphere is on the left side.

### 2.2 Tasks

#### 2.2.1 Rapid Serial Visual Presentation Task

BE was measured with the same Rapid Serial Visual Presentation Task used by Mullally et al. (2012) (see Fig. 2). Participants were informed that, on each trial, they would be viewing a picture of a simple scene twice in rapid succession, and upon the second presentation of the scene they would have to decide whether the scene was exactly the same as they had seen before, or if instead it was closer-up or farther-away. They were told that the purpose of the experiment was to determine how well people can focus their attention. After having seen an example stimulus (e.g., a pink flower on a green background, in standard and closer-up view), participants completed 24 randomly presented trials. In all cases, the initial picture comprised a single, centrally positioned object in a simple scene, presented on the computer screen for 250 msec and followed by a briefly presented (250 msec) visual noise mask. The second picture immediately followed the mask. The task was to rate the second picture relative to the first, choosing one of five options, i.e., “much closer-up”, “a little closer-up”, “the same”, “a little farther-away” or “much farther-away”. Unbeknownst to the participants, the two pictures were always identical, and thus all picture pairs should have been rated as the same (the correct answer).

**Fig. 2:**
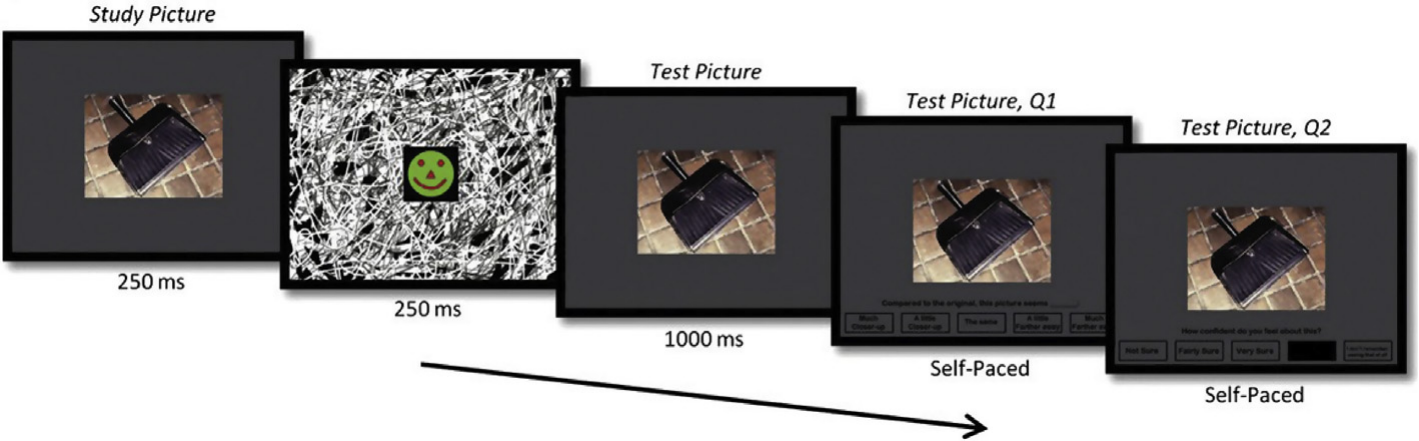
Rapid Serial Visual Presentation Task. Timeline of an example trial. First, a simple scene was presented for 250 msec, followed by a brief mask which was also presented for 250 msec. The scene image was then presented again for 1000 msec, after which a rating scale appeared underneath. The participants were asked to rate whether the two scenes were the same, or whether the second scene was a closer-up or farther-away view compared to the first scene. Participants then rated how confident they were in their decision.

The proportion of trials classified as either 'the same', 'closer-up' (collapsing across 'much closer-up' and 'a little closer-up' responses), or 'farther-away' responses (collapsing across 'much farther-away' and 'a little farther-away' responses) was calculated as the percentage of responses made in each category relative to the total number of responses made. BE is revealed by a disproportionally large number of incorrect “closer-up” responses. This is because when they initially view a scene, participants typically imagine the extended environment surrounding the scene. When this more expansive representation is subsequently compared with the second ‘test’ picture, although it is identical to the initial picture viewed only 250 msec previous, it is consistently believed to depict a closer-up scene. In addition, BE can be quantified by a mean BE score, calculated by averaging the numerical values across the 24 trials associated with the responses, i.e., “much closer-up” = −2; “a little closer-up” = −1; “the same” = 0; “a little farther-away” = +1; “much farther-away” = +2. The BE score indicates the degree of bias towards one response over another, with a mean score of ‘0’ indicating no BE effect, and negative scores reflecting BE. On each trial participants also reported how confident they were about their decision using a three-point scale (1 = “not sure”, 2 = “fairly sure”, 3 = “very sure”), and mean confidence ratings were calculated for each of the three response categories (the same, closer-up, farther-away). Given the rapid presentation of the first scene on each trial, they were also given the option to press a button to indicate that they did not see the first picture at all (5 = “don't remember”). The frequency with which this happened was very low (vmPFC patients: 4 trials; control patients: 5 trials; vmPFC healthy controls: 1 trial), as is typical in BE research, and this did not differ significantly across groups (H = 2.68; *p* = .26). These trials were discarded from subsequent analyses.

#### 2.2.2 Scene probe task

**Fig. 3:**
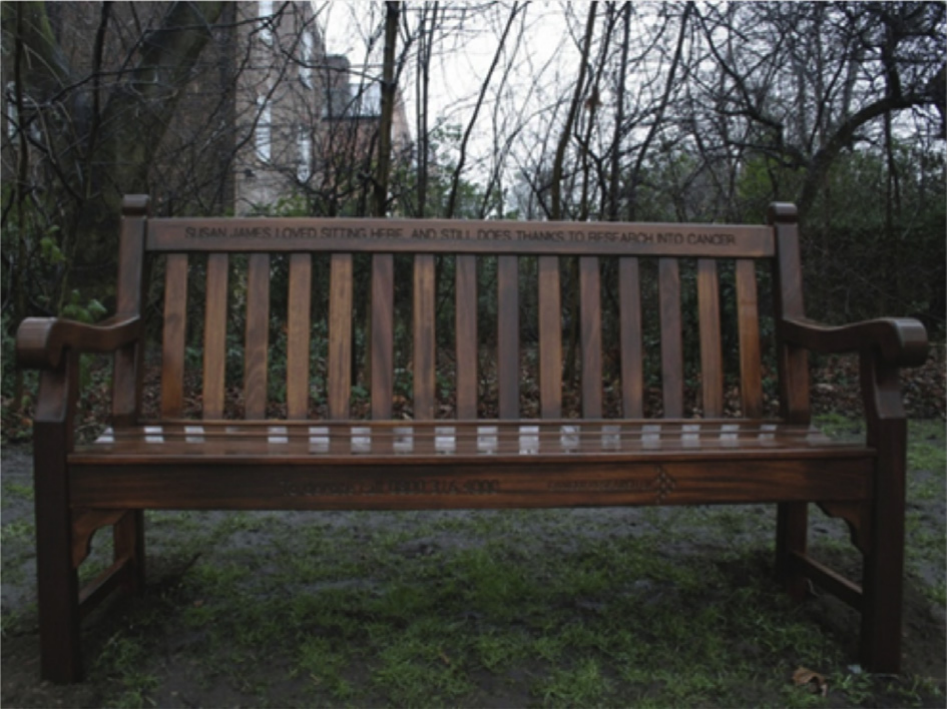
Scene probe task. The image depicts the scene stimulus used. Participants were instructed to describe this picture out loud, including the main object and background. They were then asked what would come into view if they stepped back from the current camera position.

Using a 'scene probe' task we attempted to ascertain what aspects of a scene representation might be affected by vmPFC damage. As with the hippocampal-lesioned patients in Mullally et al. (2012), a close-up photograph of a scene was displayed and remained on the screen for the duration of the task (see Fig. 3). Participants were first asked to name the main components of the scene, namely, the central object (a bench), the background (trees and houses), the type of place where the photograph was taken (a park/garden), and the predominant colors (green and brown). A score of 1 was awarded to each of the four elements of the scene correctly listed, and a score of 0 for missing elements (range 0–4). Participants were then asked to describe in as much detail as possible what the scene might be like beyond the boundaries of the current view, that is, what might come into view if they imagined taking a few steps back from the camera's current position. Participants were encouraged to use their imagination. Verbal descriptions were recorded and later transcribed. Every statement was classified as belonging to one of four categories: entities present (EP, e.g., “there is a bench”), sensory descriptions (SD, e.g., “the chair is made of wood”), spatial references (SPA; e.g., “behind the tree”), and thoughts/emotions/actions (TEA, e.g., “I felt lonely”) (Mullally et al., 2012; see also; Hassabis et al., 2007). Participants were also asked whether they were actually able to visualize the extended scene in their imagination and rate the vividness of the scene beyond the view using a 5-point scale (1 = not vivid at all … 5 = very vivid). If they were unable to visualize anything, they were given a score of 0. All descriptions were scored by author FDL (not blind to group membership), and a second rater (blind to group membership) scored 1/3 of the transcripts independently. Inter-rater reliability (separately for SD, SPA, EP, TEA), assessed with intra-class correlations (Mcgraw & Wong, 1996), was high (coefficients > .76 in all cases).

### 2.3 Data analyses

Given that in most cases the dependent variables were non-normally distributed (Kolmogorov–Smirnov d > .13, *p* < .05), behavioral data were analyzed with non-parametric tests. We compared vmPFC patients with vmPFC healthy controls and control patients. We also compared the three patient groups (vmPFC, hippocampal and control patients) directly. We did this by calculating z-scores for each patient with reference to their respective matched healthy control group (e.g., vmPFC healthy controls for vmPFC patients and control patients, and hippocampal healthy controls for hippocampal patients), and comparing z-scores across patient groups. This allowed us to control for age and education differences between the Italian and British patient cohorts. We analyzed comparisons involving the three participant groups with non-parametric Kruskall-Wallis analyses of variance (ANOVA) and conducted planned comparisons between vmPFC patients and brain-damaged and healthy controls with Mann–Whitney *z* tests - we report the exact, two-tailed, uncorrected *p* values. Where appropriate, we also report effect sizes (r) for non-normal data (based on Fritz, Morris, & Richler, 2012) where a large effect is > .5, a medium effect ∼ .3, and a small effect is ∼.1 (Coolican, 2009). All differences were considered statistically significant at *p* < .05, two-tailed.

## 3 Results

### 3.1 Rapid Serial Visual Presentation Task

#### 3.1.1 Accuracy

**Fig. 4:**
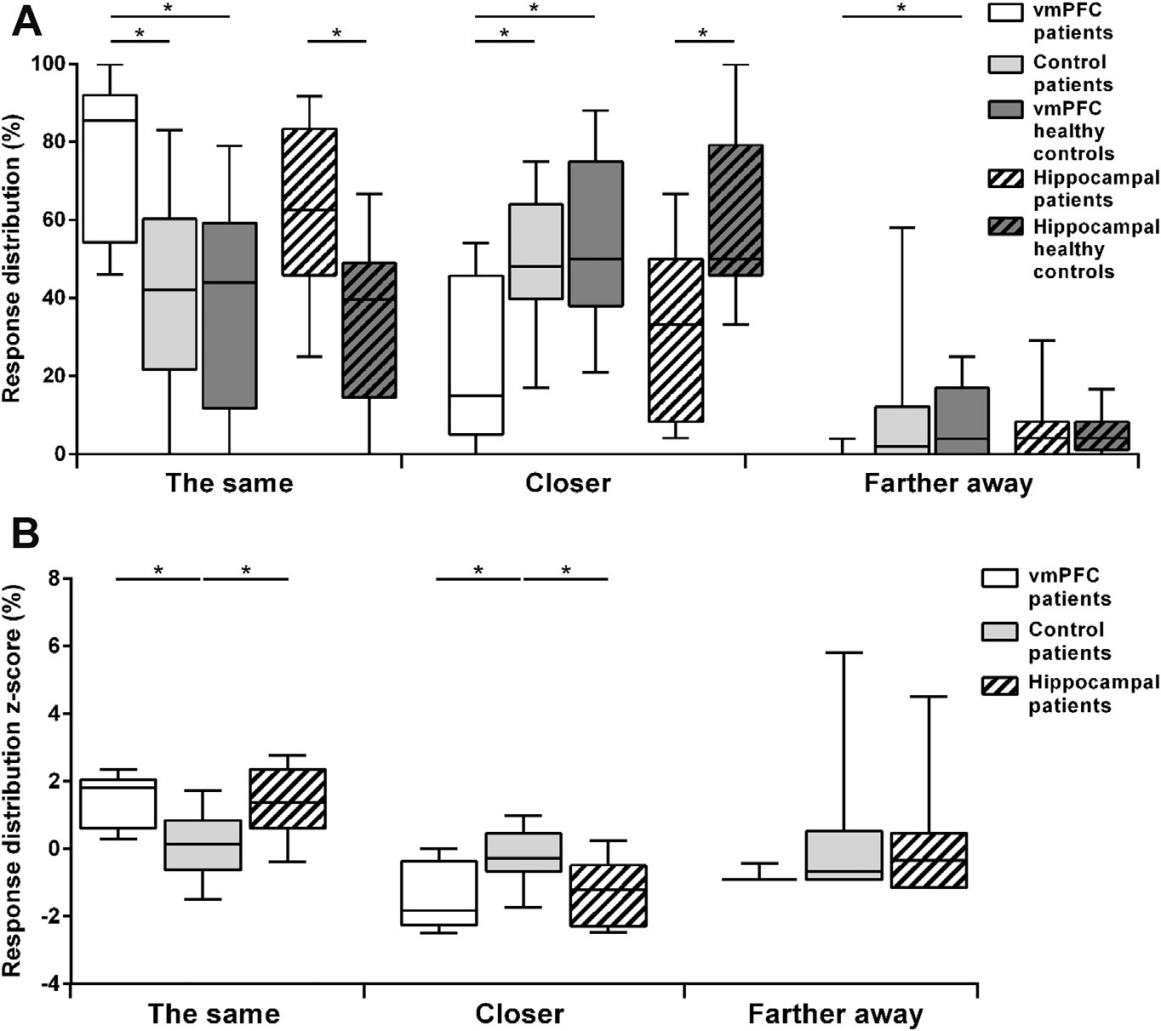
A: Box-plots of the percentage of trials classified as the same, closer-up and farther-away in the Rapid Serial Visual Presentation Task by participant group. The data and significance levels contrasting patients with hippocampal damage and their controls are from Mullally et al. (2012). B: Box-plots of z-scores for the same, closer-up, and farther-away responses for the three patient groups. Boxplots depict the median, first and third quartiles, and minimum and maximum (whiskers) of the data sets. *p < .05.

**Fig. 5:**
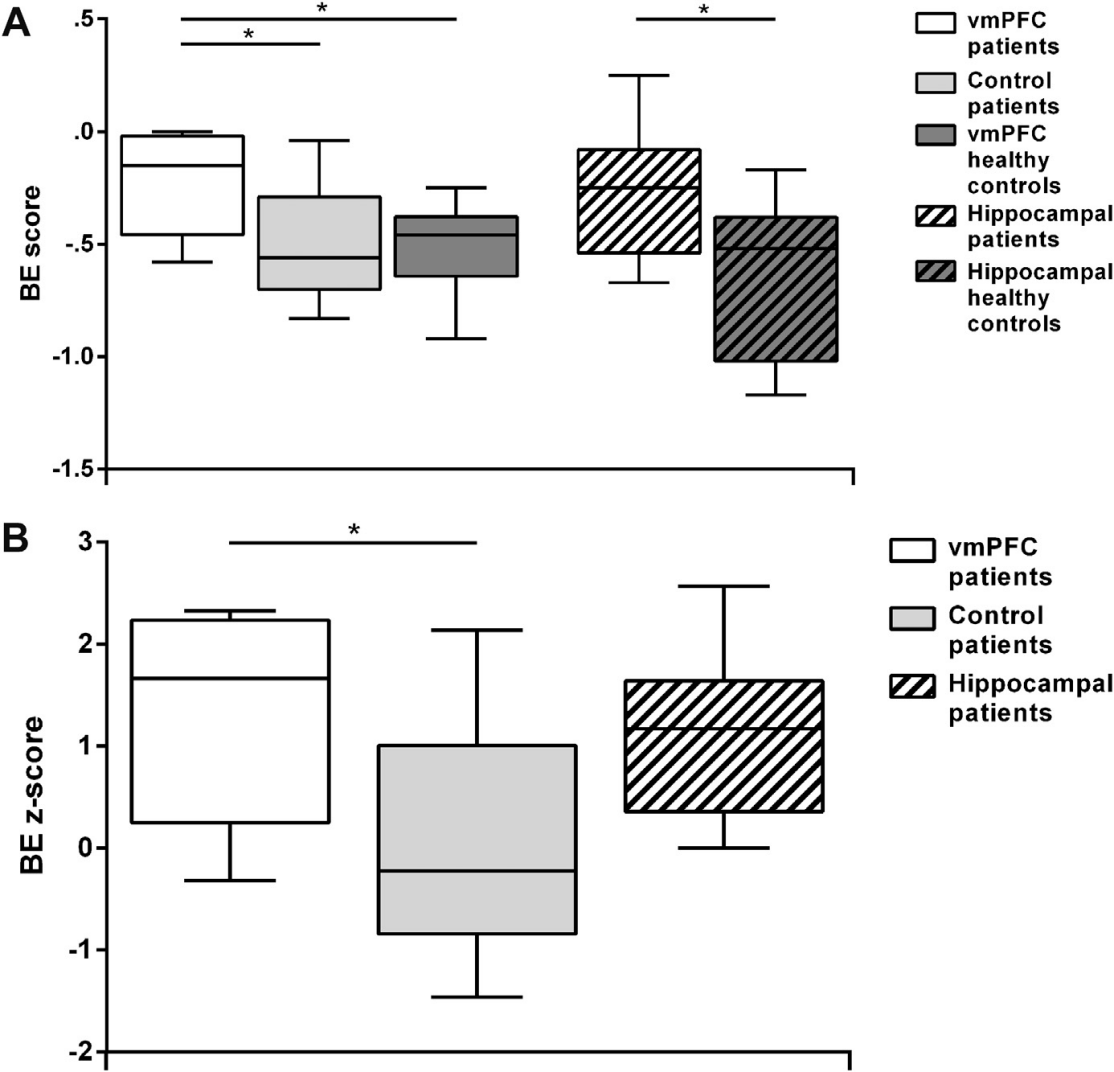
A: Box-plots of boundary extension (BE) scores by participant group. The data and significance levels contrasting patients with hippocampal damage and their controls are from Mullally et al. (2012). The more negative the score, the more BE. B: Box-plots of BE z-scores for the three patient groups. Boxplots depict the median, first and third quartiles, and minimum and maximum (whiskers) of the data sets. *p < .05.

Fig. 4A shows the boxplots of the percentage of each response type (closer-up, the same, farther-away), collapsing across the degrees of subjective distance (“much” or “a little” farther/closer), by participant group. Fig. 5A shows the boxplots of the boundary extension (BE) score by participant group. BE was apparent in all groups, as evidenced by no group selecting the correct - ‘same’- response 100% of the time. However, whereas healthy controls and control patients rated more often the second presentation of pictures as closer-up, vmPFC patients (and hippocampal patients) rated more often the pictures correctly as being the same, and thus showed attenuated BE. Statistical tests confirmed these observations. Kruskal–Wallis ANOVAs on the percentage of the same, closer-up, and farther-away responses across participant groups (vmPFC patients, control patients, vmPFC healthy controls) showed significant group differences for the same (H = 9.68, *p* = .01) and closer-up responses (H = 8.24, *p* = .02). Post hoc Mann–Whitney tests showed that vmPFC patients classified trials more often as the same compared to both vmPFC healthy controls (U = 9.00, Z = 2.75, *p* = .01, r = .65), and control patients (U = 10.00, Z = 2.66, *p* = .01, r = .63), and less often as closer-up compared to both vmPFC healthy controls (U = 11.50, Z = −2.53, *p* = .01, r = .60) and control patients (U = 12.50, Z = −2.44, *p* = .01, r = .58). There were no differences in the percentage of trials classified as the same (*p* = .91, r = .03) or closer-up (*p* = .70, r = .1) between vmPFC healthy controls and control patients. Similarly, there were significant group differences in the BE score (H = 6.11, *p* = .047), such that vmPFC patients showed a lower BE score than vmPFC healthy controls (U = 15.50, Z = 2.18, *p* = .03, r = .51) and control patients (U = 16.00, Z = 2.13, *p* = .03, r = .50), while there were no differences between the two control groups (*p* = .97, r = .01). This first set of analyses shows that vmPFC patients have a significantly reduced BE compared to both healthy and brain-damaged controls. vmPFC performance was reminiscent of that of hippocampal patients, who also showed attenuated BE (Mullally et al., 2012).

To compare BE across vmPFC patients, hippocampal patients and control patients directly, we analyzed z-scores (see Figs. 4B and 5B). Kruskal–Wallis ANOVAs on z-scores showed significant group differences in the percentage of the same (H = 8.14, *p* = .02), closer-up (H = 7.32, *p* = .03) and a strong trend in the BE score (H = 5.66, *p* = .059). There were no differences in the percentage of farther-away responses (H = 2.06, *p* = .36). Mann–Whitney post hoc tests showed that, compared to control patients, both vmPFC and hippocampal patients more often gave the correct (the same) response (vmPFC *vs*. control patients: U = 10.00, Z = 2.62, *p* = .01, r = .62; hippocampal *vs*. control patients: U = 14.00, Z = 2.00, *p* = .04, r = .49) and less often the closer-up responses (vmPFC *vs*. control patients: U = 12.50, Z = −2.40, *p* = .02, r = .57; hippocampal *vs*. control patients: U = 14.00, Z = −2.00, *p* = .04, r = .49). Importantly, there was no difference in the percentage of same, closer-up and farther-away responses between vmPFC and hippocampal patients (all *p* > .69, all r < .1), indicating that BE was reduced to a similar degree. Confirming these results, BE scores were similar between vmPFC and hippocampal patients (*p* = .60, r = .13), and were lower in vmPFC patients (U = 16.00, Z = 2.09, *p* = .04, r = .49), and hippocampal patients (U = 17.00, Z = 1.71, *p* = .09, r = .41) compared to control patients, although only the difference between vmPFC and control patients reached statistical significance. Together, the z-score results confirmed that vmPFC patients, as well as hippocampal patients, showed a reduced BE effect compared to patients with brain lesions not involving vmPFC or the hippocampus.

#### 3.1.2 Confidence

Table 2 shows confidence ratings by participant group and type of response. Kruskal–Wallis ANOVAs on confidence ratings associated with the same, closer-up, and farther-away responses across participant groups (vmPFC patients, control patients, vmPFC healthy controls) showed significant group differences in confidence ratings for closer-up responses (H = 6.84, *p* = .03), but not for the other response categories (*p* > .18 in both cases). Post hoc tests showed that vmPFC patients were less confident in their closer-up responses than vmPFC healthy controls (U = 12.00, Z = −2.24, *p* = .02, r = .54), but had similar confidence levels as the control patients (*p* = .22, r = .30). There was no significant difference in confidence for closer-up responses between control patients and vmPFC healthy controls (*p* = .07, r = .41). Comparing vmPFC patients, hippocampal patients, and control patients directly using z-scores showed there were no significant group differences in confidence associated with the same, closer-up, or farther-away responses (*p* > .07 in all cases).

**Table 2:** Confidence ratings in the Rapid Serial Visual Presentation Task by participant group.

	The same	Closer-up	Farther-away	Same z-score	Closer-up z-score	Farther-away z-score
vmPFC patients	2.60 (1.90–2.96)	2.00 (1.77–3.00)	2.00^1^	.37 (−1.26–1.16)	−1.24 (−1.98–2.00)	.33
Control patients	2.10 (1.71–2.43)	2.07 (1.86–2.60)	1.83 (1.00–2.00)	−.80 (−1.69–−.05)	−1.00 (−1.70–.71)	−.09 (−2.16–.33)
vmPFC healthy controls	2.21 (2.00–3.00)	2.29 (2.00–3.00)	2.00 (1.00–2.25)	–	–	–
Hippocampal patients	2.17 (1.36–2.88)	1.78 (1.00–2.42)	2.00 (1.00–2.00)	.53 (−1.68–2.47)	−1.14 (−2.84–.25)	.88 (−3.15–.88)
Hippocampal healthy controls	1.97 (1.33–2.62)	2.17 (1.75–3.00)	1.75 (1.00–2.00)	–	–	–

Notes. Median and range (in parentheses) of confidence scores. vmPFC = ventromedial prefrontal cortex. ^1^ = only one patient gave a farther-away response, therefore no range is reported. Confidence was rated on a three-point scale: 1 = “not sure”, 2 = “fairly sure”, 3 = “very sure”.

As a side note, given that some of the control patients had visual field deficits, one may ask whether these deficits played a role in their (normal) performance on the BE task. For example, did these patients fail to appreciate that the pictures were the same across presentations because they did not scan them completely? We therefore inspected BE scores separately for control patients with and without visual field deficits. Control patients with (M = −.47) and without visual deficits (M = −.56) had comparable BE scores (Mann–Whitney U = 7.50, Z = .68, *p* = .49, r = .22), and these were similar to those of the healthy controls (M = −.51; healthy controls *vs*. control patients with visual field deficits: U = 34.00, Z = −.10, *p* = .92, r = .02; healthy controls *vs*. control patients without visual field deficits: U = 13.50, Z = .25, *p* = .80, r = .1).

### 3.2 Scene probe task

#### 3.2.1 Contents

Upon presentation of the scene probe, all participants were able to list the main elements of the scene, with the exception of one vmPFC patient who failed to mention the location, two control patients who failed to mention in one case the location, and in the other case the colors, and two healthy controls who failed to mention the colors. The description scores were consequently very high, and comparable across participant groups (vmPFC patients: 3.88; control patients: 3.80; healthy controls: 3.80; H = .21, *p* = .90). All hippocampal patients and their controls named all of the elements (score = 4 in all cases).

**Fig. 6:**
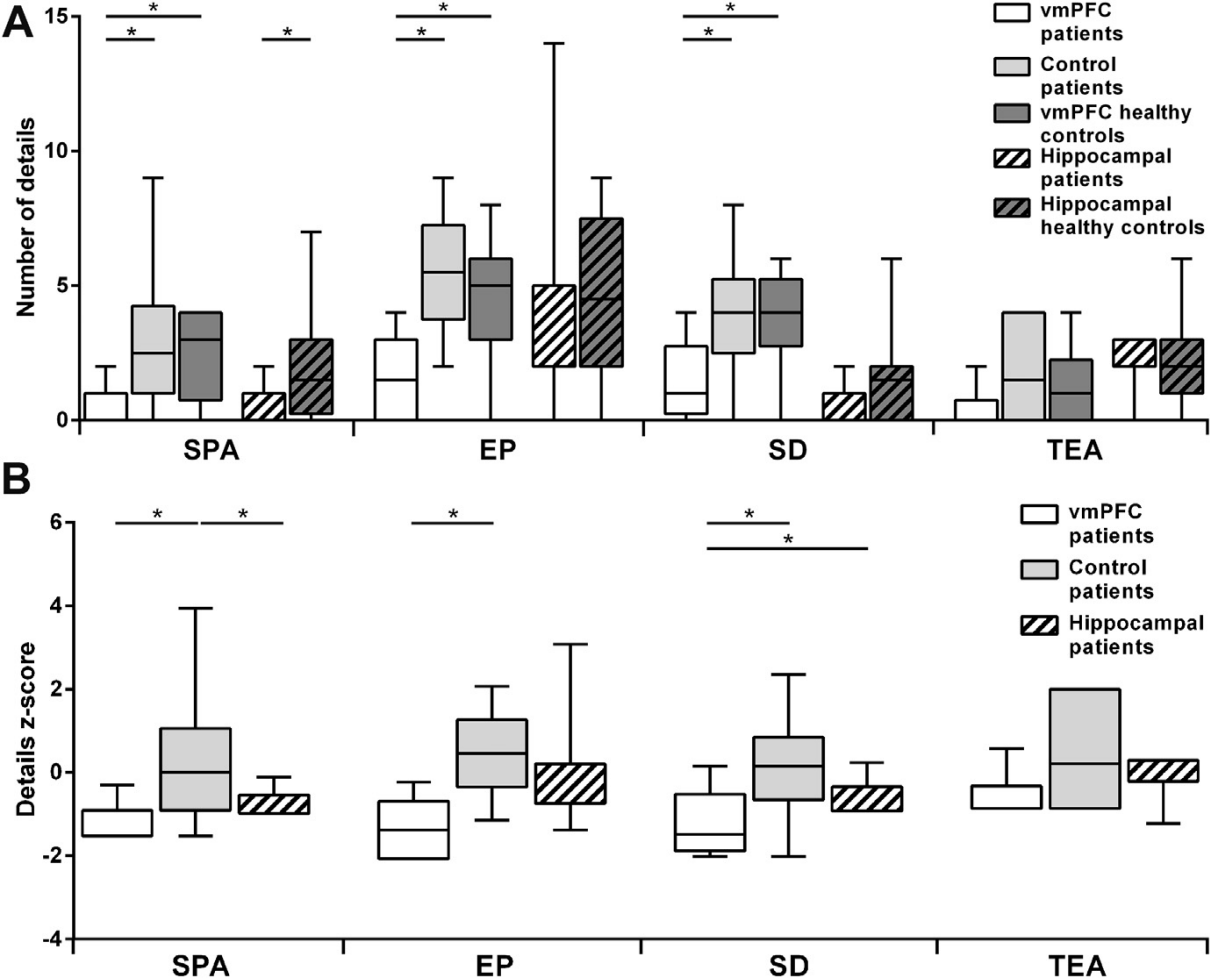
A: Box-plots of the number of constructed details in the scene probe task, with SPA = spatial references; EP = entities present, SD = sensory descriptions; TEA = thoughts/emotions/actions. The data and significance levels contrasting patients with hippocampal damage and their controls are from Mullally et al. (2012). B: Box-plots of z-scores for details produced in the scene probe task for the three patient groups. Boxplots depict the median, first and third quartiles, and minimum and maximum (whiskers) of the data sets. *p < .05.

Fig. 6A shows the boxplots of the number of details participants produced when requested to imagine what might be beyond the boundaries of the current view, by participant group and content category. Kruskal–Wallis ANOVAs across participant groups (vmPFC patients, control patients, vmPFC healthy controls) showed significant group differences in the number of entities present (EP; H = 10.73, *p* = .01), sensory descriptions (SD; H = 6.97, *p* = .03), and spatial references (SPA; H = 8.46, *p* = .01), but not thoughts/actions/emotions (TEA; *p* = .17). Mann–Whitney post-hoc tests showed that vmPFC patients produced fewer EP, SD, and SPA than both vmPFC healthy controls (EP: U = 11.00, Z = −2.58, *p* = .01, r = .61; SD: U = 14.00, Z = −2.31, *p* = .02, r = .54; SPA: U = 13.50, Z = −2.35, *p* = .02, r = .55) and control patients (EP: U = 6.50, Z = −2.98, *p* = .003, r = .70; SD: U = 15.00, Z = −2.22, *p* = .03, r = .52; SPA: U = 10.50, Z = −2.62, *p* = .01, r = .62), whereas no differences emerged between vmPFC healthy controls and control patients across content categories (*p* > .45, r < .2 in all cases). Thus, consistent with evidence of reduced BE, vmPFC patients had difficulty imagining what might be beyond the scene they were currently perceiving. Their construction of the extended scene, however, differed from that of hippocampal patients, which was specifically devoid of spatial references compared to that of the hippocampal healthy controls, while EP, SD and TEA categories were intact (see Mullally et al., 2012).

The analysis of z-scores (Fig. 6B) confirms that deficits in imagining what would be beyond the view differed between vmPFC and hippocampal patients. Kruskal–Wallis ANOVAs across participant groups (vmPFC patients, hippocampal patients, control patients) revealed significant group differences in the number of EP (H = 9.81, *p* = .01), SD (H = 7.50, *p* = .02), and SPA (H = 9.39, *p* = .01), but not TEA (*p* = .17). Both vmPFC patients (U = 10.50, Z = −2.58, *p* = .01, r = .61) and hippocampal patients (U = 13.00, Z = −2.10, *p* = .04, r = .51) produced fewer SPA than controls patients. However, vmPFC patients' reports were also impoverished with regard to EP (U = 6.50, Z = −2.93, *p* = .003, r = .69) and SD (U = 15.00, Z = −2.18, *p* = .03, r = .51) than those of controls patients, whereas hippocampal patients' reports were not (*p* > .13, r < .4 in both cases). In addition, vmPFC patients produced significantly fewer SD than hippocampal patients (U = 8.00, Z = −2.26, *p* = .02, r = .58), while SPA and EP were not significantly different between vmPFC and hippocampal patients (*p* > .12, r < .4 in both cases).

#### 3.2.2 Vividnes

Table 3 reports self-rated vividness for the imagined extended scene by patient group. A Kruskal–Wallis ANOVA on vividness ratings revealed significant differences among vmPFC patients, control patients, and vmPFC healthy controls (H = 8.73, *p* = .01), such that vmPFC patients (U = 10.00, Z = −2.67, *p* = .01, r = .63) and control patients (U = 24.00, Z = 1.97, *p* = .049, r = .44) judged the extended scene as less vivid than did healthy controls, with no difference between vmPFC patients and control patients (*p* = .28, r = .25). Hippocampal patients, too, rated the extended scenes they had imagined as less vivid than their healthy controls (Mullally et al., 2012). When we compared the three patient groups directly using vividness z-scores, we found no group differences (*p* = .43).

**Table 3:** Vividness ratings and z scores in the scene probe task by participant group.

	vmPFC patients	Control patients	vmPFC healthy controls	Hippocampal patients	Hippocampal healthy controls
Vividness	1.50 (.00–4.00)	3.00 (.00–5.00)	4.00 (3.00–5.00)	.00 (.00–5.00)	5.00 (1.66–5.00)
Vividness z-score	−4.58 (−7.22 to −.18)	−1.94 (−7.22–1.59)	–	−3.04 (−3.04–.74)	–

Notes. Median and range (in parentheses) of vividness scores. vmPFC = ventromedial prefrontal cortex. Vividness was rated using a five-point scale: 1 = not vivid at all … 5 = very vivid.

## 4 Discussion

This study investigated whether vmPFC is involved in rapid, automatic and implicit visual scene construction. To examine this, we exploited BE, a cognitive phenomenon whereby, upon viewing a scene, individuals automatically construct an internal representation of the scene that extends beyond its given borders, which is revealed by the subsequent misremembering of the extended scene instead of the original (Intraub, 2012). We contrasted performance of patients with vmPFC damage to that of control patients with (mainly) occipital lesions, healthy controls, and the hippocampal patients described in Mullally et al. (2012). The results showed that vmPFC patients have significantly reduced BE compared to healthy individuals, and the attenuation of BE was comparable to that previously observed in hippocampal-damaged patients (Mullally et al., 2012). These findings extend previous evidence of impaired scene construction in hippocampal patients by showing that vmPFC, alongside the hippocampus, is necessary to automatically construct internal representations of (extended) scenes. Importantly, we show that it is not the case that brain damage per se disrupts BE. Control patients with lesions located mainly in the occipital cortex (including those with and without visual field defects) showed BE that was similar to that of healthy controls.

The reduced BE in vmPFC (as well as hippocampal) patients cannot be attributed to a failure of memory between study and test, because it indicates that patients were in fact less prone to the error of commission made by the healthy controls, and so paradoxically outperformed healthy controls in remembering the scenes accurately. Instead, our results suggest that vmPFC (and hippocampal) patients may have a fundamental difficulty with the mental construction of scene representations. Before discussing this further, it is important to consider whether the adoption of a response heuristic or a tendency towards perseveration on the part of the vmPFC patients may have had an impact on our findings. We think it unlikely, because only one vmPFC patient always selected the 'same' response option across all trials, and the results do not change if we exclude his data from the analyses. Moreover, any tendency towards perseveration should be manifest across response types. However, the two vmPFC patients who started with a 'closer up' response did not perseverate in responding closer up on the subsequent trials, and instead switched soon to 'same' responses, as did the other vmPFC patients following their occasional 'closer up' responses. Moreover, vmPFC patients' rare errors were not random, but were in the same direction as the controls’ - they mostly consisted of closer up responses, whereas farther away responses were rare in all groups.

We propose that reduced BE in the vmPFC patients indicates a problem in the mental construction of scenes, consistent with the fact that vmPFC is part of a distributed network of brain regions engaged during scene construction (Hassabis and Maguire, 2007, Hassabis et al., 2007). That vmPFC patients have reduced BE accords with previous evidence that vmPFC patients are impaired at constructing personal past and future events, and also future events that involve other people or atemporal fictitious events (Bertossi et al., 2016a, Bertossi et al., 2016b). This is because the ability to mentally construct spatially coherent scenes is likely necessary to mentally represent and experience any complex event as an alternative to direct (perceptual) experience. In this regard, it is also notable that patients with vmPFC damage initiate fewer mind-wandering episodes compared to patients with other brain lesions or healthy controls (Bertossi & Ciaramelli, 2016; see also McCormick, Rosenthal, Miller, & Maguire, 2018b). Gathering additional convergent evidence for impaired scene construction in vmPFC patients from a BE paradigm, however, is particularly important. First, unlike hippocampal patients (Race et al., 2013, Race et al., 2015, Race et al., 2011), vmPFC patients may have impaired narrative skills (Bertossi, Candela, De Luca, & Ciaramelli, 2017). This can contribute to their poor descriptions of past and future events (Bertossi et al., 2017), but certainly not to their reduced BE because BE does not depend on language. Second, whereas previous research has investigated the voluntary and explicit construction of past and novel events in vmPFC patients (Bertossi et al., 2016a, Bertossi et al., 2016b, Kurczek et al., 2015), BE is an automatic phenomenon, and therefore hardly attributable to lack of motivation or cognitive resources in vmPFC patients.

An important question is whether the role played by vmPFC and hippocampus can be differentiated. The results from the scene probe task suggest that they can be. Both patient groups proved able to describe the relevant components of the scene in view, but failed when they were asked to imagine taking a step back from the current position and describe what might then come into view. However, the reports of vmPFC- and hippocampal-damaged patients were qualitatively different. Hippocampal patients' reports contained abnormally fewer spatial references but were not different from their controls in terms of other types of details. In contrast, vmPFC patients' reports were poor not only in terms of spatial references but also in the other types of details, including entities present and their sensory details. Thus, scenes lacked primarily spatial coherence in hippocampal patients, whereas they additionally lacked content in vmPFC patients, suggesting a more general role for vmPFC in scene construction. Of course, unlike the BE task, the scene probe task depends on language, and therefore one concern might be that a deficit in narrative ability or generally reduced verbal output may have contributed to a reduction in performance, especially in the case of vmPFC patients who produced fewer details of all types. We consider this possibility unlikely, however. First, vmPFC patients had normal verbal fluency, and no problem describing the scene that was in view. Note also that the number of different types of imagined details in the scene probe task (EP, SD, SPA, TEA) did not correlate with each other (*p* > .35 in all cases) nor with phonemic or semantic fluency (*p* > .27 in both cases). Moreover, in a previous study, we showed that impaired narrative skills do not explain poor episodic future thinking in vmPFC patients (Bertossi et al., 2017).

We propose that vmPFC and the hippocampus work in concert during scene construction, by playing different but complementary roles (McCormick, Ciaramelli, et al., 2018a). vmPFC initiates the scene construction process by predicting and then coordinating the activation of relevant schematic knowledge (e.g., the prototypical park) (Burgess and Shallice, 1996, Ghosh et al., 2014, Van Kesteren et al., 2012) which the hippocampus uses to build a first, rudimentary spatially coherent representation which includes the extended scene. The vmPFC then engages in iterations via feedback loops with neocortex and hippocampus, mediating the prediction, retrieval, monitoring, and integration of relevant elements from neocortical areas (e.g., what is typically in a park) (Moscovitch, 1992, Benoit et al., 2014, Moscovitch et al., 2016, McCormick et al., 2018a) to enrich the initial spatial sketch with appropriate details, resulting in a complex and content-rich scene. This hypothesis is in line with the finding that hippocampal patients can produce appropriate scene contents, but these appear to be “floating” in an ill-defined space, whereas vmPFC patients produce scenes poor in both spatial context and content. Of course, even though our results indicate that vmPFC is necessary to build even single scenes, its contribution is expected to be magnified during construction of complex events (McCormick, Ciaramelli, et al., 2018a). This is because events additionally entail transitions between (and hence the construction of) multiple scenes, and the predictions about, and knowledge of, how common events typically unfold (e.g., a typical day in the park), which is also supported by medial prefrontal cortex regions (Krueger, Barbey, & Grafman, 2009). This may explain why vmPFC patients are particularly poor at processing extended mental events (Bertossi et al., 2016b).

We end by acknowledging a limitation that our study has in common with many neuropsychological studies of patient populations with focal brain damage, namely, the small sample size. Given this constraint, and in order to highlight differences and commonalities between the scene construction performance of vmPFC and hippocampal patients, we have relied on uncorrected non-parametric statistics. It will, therefore, be important to corroborate these findings in future studies with larger groups of patients. We also note that, while vmPFC patients may provide task-irrelevant information during memory tasks (Ciaramelli and Ghetti, 2007, Moscovitch and Melo, 1997, Schnider and Ptak, 1999), or solutions not necessarily relevant to real-world problems (Peters, Fellows, & Sheldon, 2017), in no case did our vmPFC patients endorse schema-irrelevant information in the scene probe task (see also Bertossi et al., 2016a, Bertossi et al., 2016b). For example, no one started talking about a park and ended up talking about a beach, nor did they provide details that were inconsistent with a park. Note that here we tested vmPFC patients without spontaneous confabulation. It is possible that vmPFC patients with confabulation might provide information that deviates from schema-related knowledge (Ghosh et al., 2014, Gilboa et al., 2006) and this would be interesting to test in a future study.

To conclude, we have found that vmPFC patients show reduced BE, indicating that vmPFC is necessary for scene construction along with the hippocampus. Scenes are the backbone of complex events such that events based on familiar (compared to unfamiliar) scenes are experienced more vividly (Robin and Moscovitch, 2014, de Vito et al., 2012). Moreover, individuals who are asked to recall an event have been shown to initially set a spatial scene for the subsequent event to reside within (Robin, Wynn, & Moscovitch, 2015). Future studies will be needed to establish the precise contribution of the vmPFC, and whether it is necessary for all - prediction, retrieval, coordination, monitoring, integration of elements from neocortical areas - or just some of the processes involved in scene and event construction.

## Conflict of interest

The authors declare no conflict of interest.

## Figures and Tables

**figs1:**
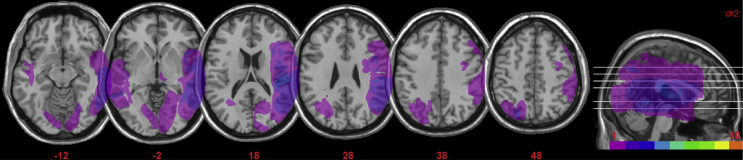


**figs2:**
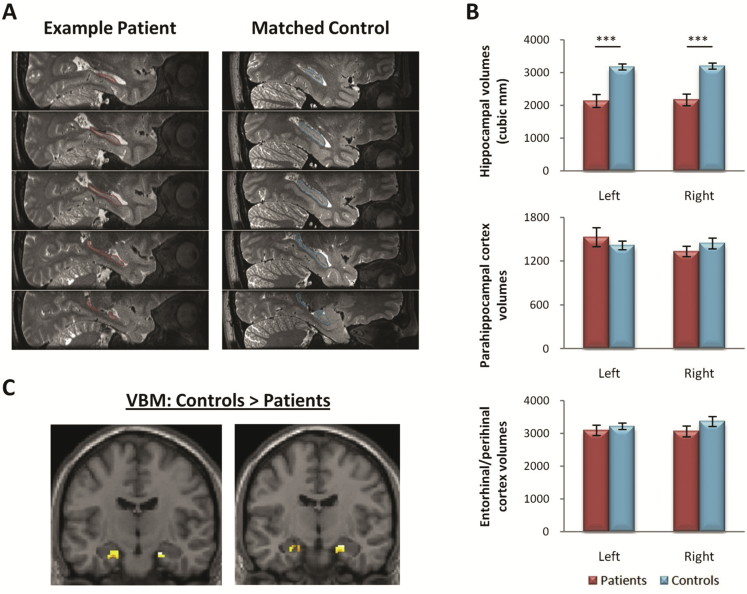


## References

[bib1] Addis D.R., Wong A.T., Schacter D.L. (2007). Remembering the past and imagining the future: Common and distinct neural substrates during event construction and elaboration. Neuropsychologia.

[bib2] Benoit R.G., Szpunar K.K., Schacter D.L. (2014). Ventromedial prefrontal cortex supports affective future simulation by integrating distributed knowledge. Proceedings of the National Academy of Sciences.

[bib3] Bertossi E., Aleo F., Braghittoni D., Ciaramelli E. (2016). Stuck in the here and now: Construction of fictitious and future experiences following ventromedial prefrontal damage. Neuropsychologia.

[bib4] Bertossi E., Candela V., De Luca F., Ciaramelli E. (2017). Episodic future thinking following vmPFC Damage: Impaired event construction, maintenance, or narration?. Neuropsychology.

[bib5] Bertossi E., Ciaramelli E. (2016). Ventromedial prefrontal damage reduces mind-wandering and biases its temporal focus. Social Cognitive and Affective Neuroscience.

[bib6] Bertossi E., Tesini C., Cappelli A., Ciaramelli E. (2016). Ventromedial prefrontal damage causes a pervasive impairment of episodic memory and future thinking. Neuropsychologia.

[bib8] Burgess P.W., Shallice T. (1996). Response suppression, Initiation and strategy use following frontal lobe lesions. Neuropsychologia.

[bib9] Chadwick M.J., Mullally S.L., Maguire E.A. (2013). The hippocampus extrapolates beyond the view in scenes: AnfMRI study of boundary extension. Cortex.

[bib10] Ciaramelli E., Ghetti S. (2007). What are confabulators' memories made of? A study of subjective and objective measures of recollection in confabulation. Neuropsychologia.

[bib11] Clark I.A., Maguire E.A. (2016). Remembering preservation in hippocampal amnesia. Annual Review of Psychology.

[bib12] Coolican H. (2009). Research methods and statistics in psychology.

[bib54] de Vito S., Gamboz N., Brandimonte M.A. (2012). What differentiates episodic future thinking from complex scene imagery?. Consciousness and Cognition.

[bib14] Fritz C.O., Morris P.E., Richler J.J. (2012). Effect size estimates: Current use, calculations, and interpretation. Journal of Experimental Psychology: General.

[bib15] Garrido M.I., Barnes G.R., Kumaran D., Maguire E.A., Dolan R.J. (2015). Ventromedial prefrontal cortex drives hippocampal theta oscillations induced by mismatch computations. Neuroimage.

[bib16] Ghosh V.E., Moscovitch M., Melo Colella B., Gilboa A. (2014). Schema representation in patients with ventromedial PFC lesions. Journal of Neuroscience.

[bib17] Gilboa A., Alain C., Stuss D.T., Melo B., Miller S., Moscovitch M. (2006). Mechanisms of spontaneous confabulations: A strategic retrieval account. Brain.

[bib18] Hassabis D., Kumaran D., Vann S.D., Maguire E.A. (2007). Patients with hippocampal amnesia cannot imagine new experiences. Proceedings of the National Academy of Sciences.

[bib19] Hassabis D., Maguire E.A. (2007). Deconstructing episodic memory with construction. Trends in Cognitive Sciences.

[bib20] Holmes C.J., Hoge R., Collins L., Woods R., Toga A.W., Evans A.C. (1998). Enhancement of MR images using registration for signal averaging. Journal of Computer Assisted Tomography.

[bib13] International Committee of Medical Journal Editors (1991). Style Matters: Statements from the vancouver group. Bmj.

[bib21] Intraub H. (2012). Rethinking visual scene perception. Wiley Interdisciplinary Reviews: Cognitive Science.

[bib22] Intraub H., Bender R.S., Mangels J.A. (1992). Looking at pictures but remembering scenes. Journal of Experimental Psychology. Learning, Memory, and Cognition.

[bib23] Intraub H., Gottesman C.V., Bills A.J. (1998). Effects of perceiving and imagining scenes on memory for pictures. Journal of Experimental Psychology. Learning, Memory, and Cognition.

[bib24] Intraub H., Richardson M. (1989). Wide-Angle Memories of Close-Up Scenes.

[bib25] Kimberg D.Y., Coslett H.B., Schwartz M.F. (2007). Power in voxel-based lesion-symptom mapping. Journal of Cognitive Neuroscience.

[bib26] Kreindel E., Intraub H. (2017). Anticipatory scene representation in preschool children’s recall and recognition memory. Developmental Science.

[bib27] Krueger F., Barbey A.K., Grafman J. (2009). The medial prefrontal cortex mediates social event knowledge. Trends in Cognitive Sciences.

[bib28] Kurczek J., Wechsler E., Ahuja S., Jensen U., Cohen N.J., Tranel D. (2015). Differential contributions of hippocampus and medial prefrontal cortex to self-projection and self-referential processing. Neuropsychologia.

[bib29] Lah S., Miller L. (2008). Effects of temporal lobe lesions on retrograde memory: A critical review. Neuropsychology Review.

[bib30] Maguire E.A., Mullally S.L. (2013). The hippocampus: A manifesto for change. Journal of Experimental Psychology. General.

[bib31] McCormick C., Ciaramelli E., De Luca F., Maguire E.A. (2018). Comparing and contrasting the cognitive effects of hippocampal and ventromedial prefrontal cortex damage: A review of human lesion studies. Neuroscience.

[bib32] McCormick C., Rosenthal C.R., Miller T.D., Maguire E.A. (2018). Mind - wandering in people with hippocampal damage. Journal of Neuroscience, in press.

[bib33] Mcgraw K.O., Wong S.P. (1996). Forming inferences about some intraclass correlation coefficients forming inferences about some intraclass correlation coefficients. Psychological Methods.

[bib34] Moscovitch M. (1992). Memory and working-with-memory: A component process model based on modules and central systems. Journal of Cognitive Neuroscience.

[bib35] Moscovitch M., Cabeza R., Winocur G., Nadel L. (2016). Episodic memory and Beyond: The Hippocampus and neocortex in transformation. Annual Review of Psychology.

[bib36] Moscovitch M., Melo B. (1997). Strategic retrieval and the frontal lobes: Evidence from confabulation and amnesia. Neuropsychologia.

[bib37] Mullally S.L., Intraub H., Maguire E.A. (2012). Attenuated boundary extension produces a paradoxical memory advantage in amnesic patients. Current Biology.

[bib38] Park S., Intraub H., Yi D.J., Widders D., Chun M.M. (2007). Beyond the edges of a view: Boundary extension in human scene-selective visual cortex. Neuron.

[bib40] Peters S.L., Fellows L.K., Sheldon S. (2017). The ventromedial frontal lobe contributes to forming effective solutions to real-world problems. Journal of Cognitive Neuroscience.

[bib41] Quinn P.C., Intraub H. (2007). Perceiving “outside the box” occurs early in development: Evidence for boundary extension in three- to seven-month-old infants. Child Development.

[bib42] Race E., Keane M.M., Verfaellie Mieke (2011). Medial temporal lobe damage causes deficits in episodic memory and episodic future thinking not attributable to deficits in narrative construction. Journal of Neuroscience.

[bib43] Race E., Keane M.M., Verfaellie M. (2013). Living in the moment: Patients with MTL amnesia can richly describe the present despite deficits in past and future thought. Cortex.

[bib44] Race E., Keane M., Verfaellie M. (2015). Sharing mental simulations and stories: Hippocampal contributions to discourse integration. Cortex.

[bib45] Robin J., Moscovitch M. (2014). The effects of spatial contextual familiarity on remembered scenes, episodic memories, and imagined future events. Journal of Experimental Psychology. Learning, Memory, and Cognition.

[bib46] Robin J., Wynn J., Moscovitch M. (2015). The spatial Scaffold : The effects of spatial context on memory for events. Journal of Experimental Psychology. Learning, Memory, and Cognition.

[bib47] Schnider A., Ptak R. (1999). Spontaneous confabulators fail to suppress currently irrelevant memory traces. Nature Neuroscience.

[bib48] Seamon J., Schlegel S., Hiester P., Landau S., Blumenthal B. (2002). Misremembering pictured objects: People of all ages demonstrate the boundary extension illusion. The American Journal of Psychology.

[bib49] Spanò G., Intraub H., Edgin J.O. (2017). Testing the “boundaries” of boundary Extension: Anticipatory scene representation across development and disorder. Hippocampus.

[bib50] Spinnler H., Tognoni G. (1987). Standardizzazione e taratura italiana di test neuropsicologici. Ital.J.Neurol.Sci.

[bib51] Svoboda E., McKinnon M.C., Levine B. (2006). The functional neuroanatomy of autobiographical memory: A meta-analysis. Neuropsychologia.

[bib52] Talairach J., Tournoux P. (1988). Co-planar stereotaxic atlas of the human brain. 3-Dimensional proportional System: An Approach to Cerebral Imaging.

[bib53] Van Kesteren M.T.R., Ruiter D.J., Fernandez G., Henson R.N. (2012). How schema and novelty augment memory formation. Trends in Neurosciences.

[bib55] Zeidman P., Maguire E.A. (2016). Anterior hippocampus: The anatomy of perception, imagination and episodic memory. Nature Reviews Neuroscience.

