# Supplementary material for: Boundary extension is attenuated in patients with ventromedial prefrontal cortex damage
Source: Cortex. 2018 Nov;108:1–12. doi: 10.1016/j.cortex.2018.07.002 (PMC6238077; doi:10.1016/j.cortex.2018.07.002)
Supplement: Multimedia component 1 [file mmc1.docx]

| **ID** | **Sex** | **Age**  **(yrs)** | **H’ness** | **Education Level** | **Aetiology** | **Chronicity (yrs)** | **Full Scale IQ** | **Matrix**^a^ **Reasoning** | **Retrograde Amnesia (yrs)** | **Complex Figure**^b^ | **Recognition** | **Recall** |
| --- | --- | --- | --- | --- | --- | --- | --- | --- | --- | --- | --- | --- |
| A | M | 63 | R | University | LE | 6 | 99 | 11 | 10 | 10 | Unimpaired | Impaired |
| B | M | 40 | R | A-Levels | Anoxia | 21 | 112 | 16 | <1 | 2 | Unimpaired | Impaired |
| C | M | 37 | R | University | LE | 3.5 | 107 | 11 | ~25 | 1 | Impaired | Impaired |
| D | F | 32 | R | University | LE | 7 | 109 | 13 | 10 | 4 | Borderline | Impaired |
| E | M | 38 | R | GCSE | LE | 3.5 | 99 | 8 | 1 | 1 | Impaired | Impaired |
| F | F | 40 | R | University | Unknown | 22 | 105 | 14 | 18 | 5 | Unimpaired | Impaired |
| G | F | 40 | L | University | Unknown | 24 | 105 | 13 | 16 | 5 | Unimpaired | Impaired |

**Supplementary Table S1**

Summary of hippocampal-damaged patients’ details.

H’ness = handedness; Chronicity = number of years since the illness/incident precipitating the hippocampal damage/memory loss; A-Levels = school examinations taken at the point of leaving secondary school ~18 years of age; GCSE = school examinations taken ~14-16 years of age; LE = limbic encephalitis. ^a^Scaled score, matrix reasoning subtest of the WAIS-III; ^b^Complex Figure (Rey-Osterrieth /BMIBP) delayed recall (percentile score). See Mullally et al. (2012) for more details.

**Supplementary Table S2**

Mean percentage of accurate responses in visual detection and visual search tasks (Bolognini, Rasi, Coccia, & Làdavas, 2005; Passamonti, Bertini, & Làdavas, 2009) attained by control patients with hemianopia (N = 7).

| Test | Visual detection: eye movements not allowed | | Visual detection: eye movements allowed | | Visual search: letters | Visual search: numbers |
| --- | --- | --- | --- | --- | --- | --- |
|  | **R** | **L** | **R** | **L** |  |  |
| Control patients with left hemisphere lesions | 34* | 92 | 86 | 97 | 100 | 100 |
| Control patients with right hemisphere lesions | 98 | 58* | 98 | 92 | 89 | 100 |

R = right hemifield, L = left hemifield. Asterisks highlight a pathological performance.

Bolognini N., Rasi F., Coccia M. and Làdavas E., Visual search improvement in hemianopic patients after audio-visual stimulation, Brain 128 (12), 2005, 2830–2842 https://doi.org/10.1093/brain/awh656.

Passamonti C., Bertini C. and Làdavas E., Audio-visual stimulation improves oculomotor patterns in patients with hemianopia, Neuropsychologia 47 (2), 2009, 546–555 https://doi.org/10.1016/j.neuropsychologia.2008.10.008.

**Supplementary Figure S1**


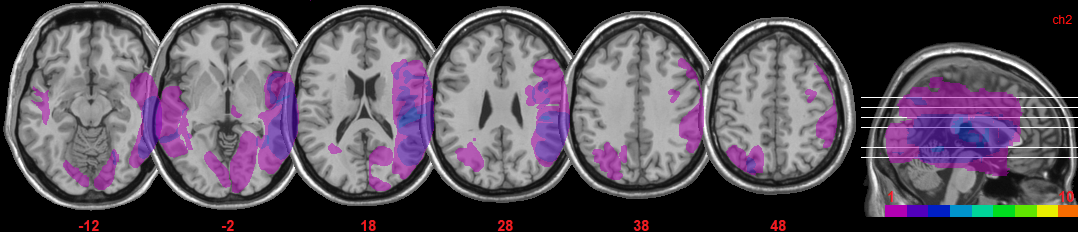


Representative axial slices and cumulative midsagittal view of the standard Montreal Neurological Institute brain showing the extent of lesion overlap in control patients. The white horizontal lines on the sagittal view are the positions of the axial slices, and the red numbers below the axial views are the z coordinates of each slice. The color bar indicates the number of overlapping lesions. Maximal overlap occurred in BA 21-22, 37, 19. The left hemisphere is on the left side.

**Supplementary Figure S2**


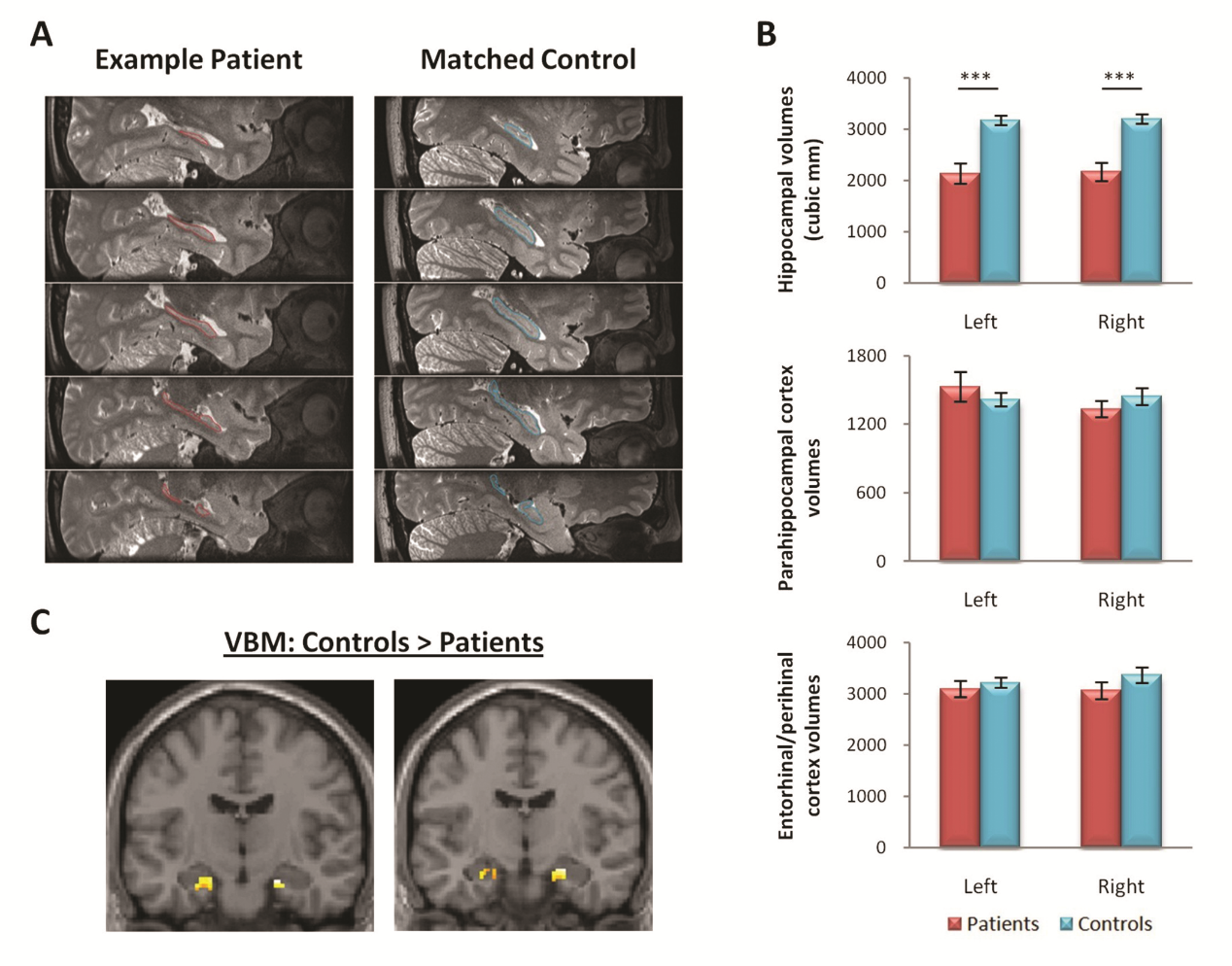


Lesion characterisation in the hippocampal-damaged patients. (A) An example high-resolution T2-weighted scan from a hippocampal patient and one of their matched controls. The outer boundary of the hippocampus is illustrated in red for the patient and blue for the control. (B) Volumetric measurements were extracted from the manually segmented medial temporal lobe regions. Significant reductions in hippocampal volume were observed in the patient group relative to the matched control group (***p < 0.001). By contrast, no significant volume differences were found in the parahippocampal or entorhinal/perirhinal cortices. (C) An automated VBM analysis was performed to identify regions, at a whole-brain level, where grey matter volume differed significantly between the control and patient groups. Selective reductions in grey matter volume were observed in the patients, relative to the control group, only in the left and right hippocampus (images are displayed at P < 0.05 uncorrected).

**Supplementary Material - Example Descriptions**

Example responses on the scene probe task ("If you were taking the picture and you took a few steps backwards, what do you think would come into view?").

*vmPFC patient n.6:* “If it [the picture] expands enough, there will be some people, children playing, there are some branches, quite big, I am not sure if it is possible to see whether it has any fruit, but I do not think it does.”

*Control patient n.7:* “I imagine that if I get farther, there could be a small artificial lake in front of the bench, where there might be small colored carps, or some water lilies or some water hyacinths, anybody can sit on the bench to relax, watching the water. On one side there could be a fountain, which would delimit this lake, and on the other side there could be a small flatter and a free area where kids play and parents, seated on the bench, watch them. Farther away, you can see better the roof of the house, the visible part seems like a skylight, I imagine the high part as quite pointy, tilted, similar to a Nordic environment, and the attic windows and another chimney on the left are also visible.”

*Healthy control n.7:* “I could see a trash can on one side, a lamppost, it's a lamppost working also as a trash can. I could also see a Coke can on the ground. Maybe the border of a pavement, cobbled with bricks, like cement covered with stones. If I went farther, I would see a bigger portion of the house, maybe one more window. On this side there could be a window, and on the other side there could be more plants.”
